# Aspirin is as effective as low molecular weight heparins in preventing symptomatic venous thromboembolism following arthroscopic anterior cruciate ligament reconstruction

**DOI:** 10.1186/s12891-024-07282-8

**Published:** 2024-02-19

**Authors:** Hamidreza Yazdi, Arvin Eslami, Ali Torkaman, Omid Elahifar, Amir Kasaeian, Shaya Alimoghadam, Rojina Alimoghadam, Mansour Abolghasemian

**Affiliations:** 1 Bone and Joint Reconstruction Research Center, Department of Orthopedics; Department of Orthopedic, School of Medicine; Firoozgar Clinical Research Development Center (FCRDC), Iran University of Medical Sciences, Tehran, Iran; 2 Digestive Diseases Research Center, Digestive Diseases Research Institute; Research Center for Chronic Inflammatory Diseases; Clinical Research Development Unit, Shariati Hospital, Tehran University of Medical Sciences, Tehran, Iran; 3 University of Alberta, Alberta, Canada

**Keywords:** Anterior cruciate ligament reconstruction, Venous thromboembolism, Prophylaxis, Aspirin, Low molecular weight heparin

## Abstract

**Objective:**

The optimal agent for thromboprophylaxis following arthroscopic anterior cruciate ligament reconstruction (ACLR) remains unclear, particularly in patients with a low baseline risk for venous thromboembolism (VTE). This retrospective cohort study aims to compare the effectiveness and safety of aspirin versus low molecular weight heparins (LMWHs) in this specific patient population.

**Methods:**

We analyzed data from patients who underwent ACLR between March 2016 and March 2021, focusing on those with a low risk for VTE. High-risk individuals, identified by factors such as cardiac disease, pulmonary disease, diabetes mellitus, previous VTE, inflammatory bowel disease, active cancer, and a BMI > 40, were excluded (*n* = 33). Our approach included a thorough review of medical charts, surgical reports, and pre-operative assessments, complemented by telephone follow-up conducted over a 3-month period by a single investigator. We assessed the incidence of symptomatic VTE, including deep vein thrombosis and pulmonary thromboembolism, as the primary outcome. The secondary outcomes included to complications related to the surgery and thromboprophylaxis. Statistical analysis included descriptive statistics, univariate logistic regression models, and calculations of incidence rates.

**Result:**

In our study, 761 patients (761 knees) were included, with 458 (60.18%) receiving aspirin and 303 (39.82%) receiving LMWH. The two groups showed no significant differences in demographic factors except for age. The incidence of VTE was reported at 1.31% (10 individuals). Specifically, five patients in the aspirin group (1.09%) and five patients in the LMWH group (1.65%) developed a symptomatic VTE event (*p* = 0.53). Additionally, the two groups did not significantly differ in terms of other complications, such as hemarthrosis or surgical site infection (*p* > 0.05). Logistic regression analysis revealed no statistically significant difference in VTE risk between the two groups.

**Conclusion:**

This study, focusing on isolated ACLR in patients with a low baseline risk for venous thromboembolism, demonstrated that aspirin is equally effective as low molecular weight heparins for VTE prophylaxis following this surgery.

**Level of Evidence:**

III

**Supplementary Information:**

The online version contains supplementary material available at 10.1186/s12891-024-07282-8.

## Background

Arthroscopic anterior cruciate ligament reconstruction (ACLR) is one of the most common orthopedic operations performed worldwide. In recent years, there has been an increase in the number of patients undergoing this surgery, particularly among women, those older than 40 or younger than 20 [1]. One of the most concerning complications of this procedure is the development of venous thromboembolism (VTE), including deep vein thrombosis (DVT) and pulmonary embolism (PE), with studies reporting an incidence of 0.2 to 2.1% for symptomatic deep venous thrombosis (DVT), and 0.4–2.87% for VTE [2–8]. In a prospective study, a cohort of 55 patients who did not receive prophylaxis was observed. The study reported a significant incidence of asymptomatic DVT at 16.4% and PE at 7.3% following ACLR [9]. 

Historically, it was controversial whether to use chemoprophylaxis for VTE after ACLR [10]. While some studies suggest pharmacologic thromboprophylaxis for ACLR in patients with risk factors such as advanced age, tobacco use, oral contraceptive pills (OCP) use, and long duration of surgery [3, 6, 7, 10–15], prospective studies have found clinically important differences in the VTE rate when a routine thromboprophylaxis policy is followed [16, 17].

The recent International Consensus Meeting (ICM) supported the idea of not providing routine VTE prophylaxis after ACLR. However, it is important to note that no study with a high level of evidence has definitively advocated against chemoprophylaxis [18]. In contrast, in a 2022 expert panel, 38.8% of the panel members agreed on the need for routine chemoprophylaxis after ACLR [10]. Furthermore, two studies with levels of evidence one and two have suggested the superiority of chemoprophylaxis [16, 17].

There is no consensus on the optimal chemoprophylaxis regimen after ACLR, a point highlighted by the ICM [18]. Low-molecular-weight heparins (LMWH) have been found to be effective to prevent VTE following ACLR in patients with low bleeding risk [16, 17]. More recently, some studies have suggested the routine use of aspirin (ASA) after arthroscopic knee surgeries in general [10, 19–21].

To our knowledge, there are no studies specifically comparing ASA with LMHWs in patients undergoing ACLR, particularly in those considered at low risk for VTE. This study aims to address this gap in the literature by focusing on patients with a low baseline risk of VTE. The intention is to provide guidance to surgeons who routinely administer chemoprophylaxis post-ACLR.

Our primary objective is to compare the efficacy of LMWH and ASA in preventing symptomatic VTE following ACLR, with the hypothesis that LMWH would reduce the incidence of symptomatic VTE compared with ASA. Additionally, our secondary objective is to evaluate and compare the safety profiles of LMWH and ASA agents in these patients.

## Materials and methods

This retrospective cohort study was conducted at two medical centers after obtaining approval from an ethics review board. Patients who underwent ACLR surgery between March 2016 and March 2021 at our institution by two fellowship-trained sports surgeons were identified using CD-9 code 204,920. This code was used to locate individuals who underwent ACLR surgery. Following the identification, a detailed review of medical records was conducted to confirm eligibility based on our inclusion criteria: Having at least three months of follow-up (Attrition rate = 0%), and being aged between 18 and 45 at the time of surgery. A total of 1022 patients were confirmed to meet these criteria and were included in the study. This process was the exclusive method for identifying potential study participants. Exclusion criteria included techniques other than autogenous hamstring tendon graft, patients with a high baseline risk for VTE determined by established criteria, those undergoing concomitant surgery in the ipsilateral knee or other sites of the body, and individuals who received chemoprophylaxis other than ASA or LMWH or none. The decision to exclude treatments other than ASA, LMWH was made to ensure a focused comparison between these two agents in our study on VTE prophylaxis following ACLR. This choice was intended to enhance clarity and specificity in our analysis. Including patients on alternative chemoprophylaxis could have introduced variability, potentially confounding our results and diminishing the relevance of our findings to contemporary clinical practice. By exclusively investigating ASA and LMWH, we aimed to offer precise insights into their comparative efficacy and safety, thereby bolstering the internal validity of our study. Concomitant surgeries introduce variables that could impact outcomes, including VTE, infection rate, postoperative pain, and rehabilitation. By exclusively studying isolated ACLR, we aimed to minimize confounding factors, enhancing the internal validity of our findings on the efficacy and safety of aspirin versus low molecular weight heparins for VTE prophylaxis. This decision strengthens the clarity and applicability of our work to clinical practice.

The determination of high baseline risk for VTE was based on recognized factors, such as advanced age, obesity, prior history of VTE, and comorbid conditions such as cardiac disease, pulmonary disease, Diabetes Mellitus (DM), Inflammatory Bowel Disease (IBD), active cancer, and BMI > 40, which have been consistently associated with an increased risk of VTE in the relevant medical literature [22]. These criteria were utilized to guide the exclusion of high-risk patients from the study in order to focus on individuals at low baseline risk for VTE.

One of the two surgeons routinely used ASA, except in high-risk patients, while the other preferred enoxaparin for VTE prophylaxis. High-risk patients under the care of the surgeon who preferred ASA had received LMWH and were consequently excluded. This exclusion was essential to minimize potential bias in the study.

Demographical data including age, gender, body mass index (BMI), in addition to the anticoagulant regimen, and relevant clinical findings at each follow-up visit were collected. Patients were typically followed at 2, 6, and 12 weeks postoperatively, and the presence of symptomatic VTE was assessed clinically. Color Doppler ultrasound is a well-established, non-invasive method for detecting DVT in the extremities, often considered a first-line diagnostic tool due to its safety and availability. However, its effectiveness in detecting PE is limited. On the other hand, Chest CT angiography is commonly employed for diagnosing PE, offering high sensitivity and specificity, making it the gold standard for PE diagnosis [23, 24]. 

In our study, we employed these diagnostic methods based on the patients’ clinical presentation. Color Doppler ultrasound was primarily used to detect DVT in the lower extremities, while chest CT angiography was employed for diagnosing PE when clinically indicated.

The rehabilitation protocol was consistent across both groups and involved initial protected weight-bearing and range-of-motion exercises commencing the day after surgery. Patients progressed to gradual weight-bearing and maintained as-tolerated weight-bearing with two crutches for one month postoperatively. Subsequently, patients transitioned to walking without crutches with full weight bearing.

Telephone interviews were conducted over a 3-month time period by the same healthcare professional to ensure consistency in data collection. During the interview, the patient was systematically questioned about specific post-operative experiences. The questionnaire included inquiries regarding [25]:

- Calf or thigh pain (Yes/No).
- Calf or thigh swelling (Yes/No).
- Chest pain episodes (Yes/No).
- Shortness of breath episodes (Yes/No).
- Diagnosis of clot formation in either of the legs (Yes/No).
- Diagnosis of lung embolism (Yes/No).
- Any bleeding from/around the wound requiring medical attention (Yes/No).
- Any abnormal bleeding in other areas of the body requiring medical attention (Yes/No).

Additionally, patients were asked about other complications, including wound discharge, wound infection, a history of irrigation and debridement (I&D) after the initial surgery, and deep knee infections. To ensure data accuracy, we cross-referenced subjective phone interview reports, with medical chart reviews. After interviews, we meticulously examined postoperative notes and follow-up records. Positive findings were documented and compared with phone interview reports. This process confirmed the reliability of patient-reported outcomes. Discrepancies were resolved by prioritizing clinical documentation and further investigating discrepancies. This method ensured a comprehensive and accurate assessment of postoperative complications, enhancing study validity.

The study population was divided into two groups based on the anticoagulant agent they received after the surgery, either ASA (100 mg per day, orally for 3 weeks) or LMWH (Enoxaparin, 40 mg per day, subcutaneously for 2 weeks), starting on the day after surgery. The data was extracted from our prospectively-collected database and confirmed with phone-call interviews with the patients.

The primary objective of this study was to compare the rates of VTE between the groups. The secondary objectives included assessing bleeding-related outcomes, such as hemarthrosis, and identifying the risk factors for VTE development after arthroscopic ACLR. Diagnostic criteria for secondary outcomes were based on clinical evaluation, including symptoms and signs like pain, swelling, and clinical assessment for hemarthrosis.

Furthermore, during the phone interviews, we posed subjective inquiries regarding the presence of wound discharge, wound infection, the necessity for I&D, and deep joint infection. All this subjective data was cross-referenced with the patients’ chart reviews.

### Statistical analysis

Descriptive statistics were expressed as number (percentage) for categorical variables and mean (standard deviation (SD)), median (interquartile range (IQR)) for continuous variables. Demographic factors, the rate of VTE events and other complications were statistically compared between the groups.

Homogeneity among the two groups was evaluated using Chi-square or Fisher exact test for categorical variables and *t-test* or Wilcoxon rank-sum test (Mann-Whitney test) for continuous variables. Binary logistic regression was performed to assess the effect of risk factors on the likelihood of developing VTE. Age, gender, BMI, and anticoagulant medication had four separate univariate models fitted for them, and because all of their *P*-values were greater than 0.2, we decided not to run a multivariate logistic regression model [26, 27]. Odds ratios, 95% confidence intervals, and *p*-values were provided for each model. A significance level of 0.05 was set for all analyses. The analyses were conducted using Stata software (StataCorp. 2019. *Stata Statistical Software: Release 16*. College Station, TX: StataCorp LLC).

## Results

A total of 1,022 patients were identified, out of which 1,005 patients were successfully contacted and completed the data forms and telephone interviews (98.33%). One hundred twenty-one patients were excluded due to concomitant surgery on the knee. Fifty-three patients were operated on using other techniques or with an allograft, in addition to twenty-five patients who had arbitrarily discontinued taking anticoagulants were excluded. Sixteen patients of the surgeon who preferred ASA that were deemed high-risk had received LMWH and were also excluded. The demographic characteristics of the excluded patients closely resembled those of the included study cohort, with a predominant age range of 18 to 45 years and a male majority.

**Fig. 1:**
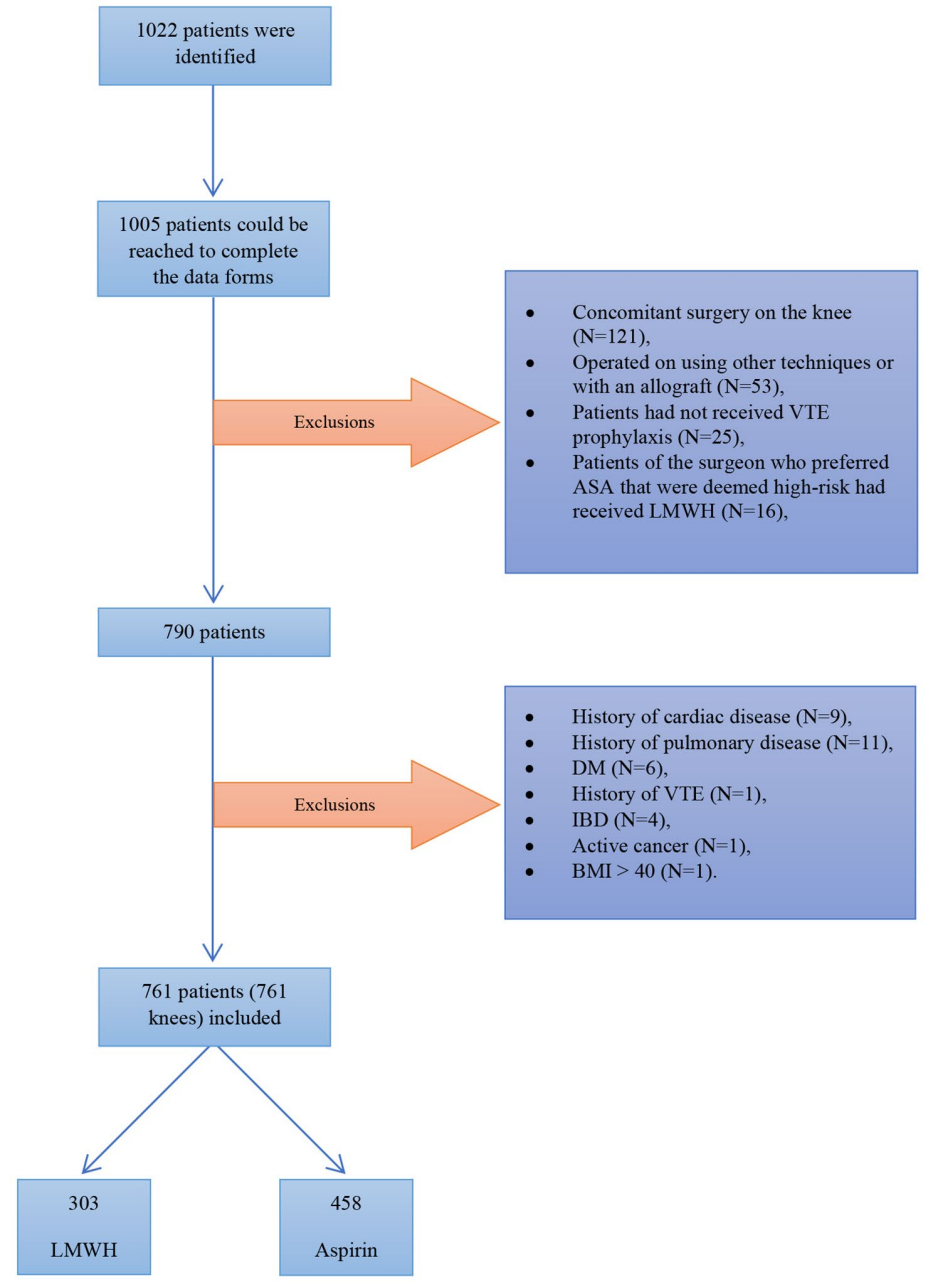
Flowchart of included patients

This left us with 790 patients. Then, high-risk patients for VTE, including those with a history of cardiac disease (*N* = 9), pulmonary disease (*N* = 11), DM (*N* = 6), previous history of VTE (*N* = 1), IBD (*N* = 4), active cancer (*N* = 1), and BMI > 40 (*N* = 1) were excluded (Fig. 1). Finally, 761 patients (761 knees) were included in the study (details shown in the appendix).

There were 458 (60.18%) and 303 (39.82%) patients in the ASA and LMWH groups, respectively. All patients had been followed for a minimum of three months postoperatively. Table 1 summarizes the baseline characteristics of the patients before the surgery. The age difference was significant, with a mean of 1.22 years between groups (Aspirin: 29.3 ± 8.4 years, LMWH: 30.5 ± 7.1 years, *p* = 0.04). No significant differences were observed in sex (*p* = 0.22), BMI (*p* = 0.80), smoking (*p* = 0.81), or cause of injury (*p* = 0.22). Table 2 describes the complications occurring within the three months of surgery in each of the groups, indicating no statistically significant differences in any of the items. In our study, VTE incidence, reported at 1.31% (10 individuals), was calculated per knee. This incidence was evenly distributed between the two treatment groups, with 5 patients (1.09%) in the Aspirin group and 5 patients (1.65%) in the Low Molecular Weight Heparin (LMWH) group, yielding a *p*-value of 0.53. These findings were further verified during phone interviews, confirming the VTE events among patients.

**Table 1:** Baseline characteristics of the patients of the two groups

		Aspirin		LMWH	*P*-value
Treatment		458 (60.2%)		303 (39.8%)	
Sex	Male	401 (87.5%)		274 (90.4%)	0.22
Age(y)		29.3 ± 8.4		30.5 ± 7.1	0.04
BMI(Kg/m^2^)		25.1 ± 3.6		25.4 ± 3.7	0.80
Smoking	Yes	50 (10.9%)		31 (10.2%)	0.81
Injury Cause	Sport	416 (90.8%)		264 (87.1%)	0.22
	Falling	27 (5.9%)		23 (7.6%)	
	Accident	13 (2.8%)		11 (3.6%)	
	Direct Trauma	2 (0.4%)		5 (1.6%)	

Categorical- and Continuous variables are presented in *number (percent)* and *mean* ± standard deviation formats, respectively. y: years, Kg: weight in kilograms, m: height in meters.

**Table 2:** Complication profile

			Aspirin	LMWH	*P*-value
Treatment			458 (60.2%)	303 (39.8%)	
VTE	DVT		3 (0.6%)	4 (1.3%)	0.72
	PTE		1 (0.2%)	0	
	Both		1 (0.2%)	1 (0.3%)	
	Total		5 (1.1%)	5 (1.6%)	0.53
Other Complications	Wound Discharge		22 (4.8%)	15 (4.9%)	0.97
	Wound Infection		14 (3.1%)	7 (2.3%)	0.65
	Need for I&D		5 (1.1%)	6 (2.0%)	0.36
	Deep Knee Infection		4 (0.9%)	5 (1.6%)	0.49
	Swelling & Hemarthrosis		19 (4.1%)	11 (3.6%)	0.85

VTE: venous thromboembolism, PTE: pulmonary thromboembolism, I&D: irrigation & debridement

Events are presented as *numbers (percentages)*

Of note, only two of the DVTs in each group were proximal to the knee. There was one case of symptomatic pulmonary embolism in the aspirin group that was treated pharmacologically with complete clinical recovery. Only three of the patients who reported noticeable leg swelling in the postoperative period during the phone interview had not been assessed with Doppler ultrasound investigation (0.39%) (2 in the ASA group and 1 in the LMWH group). Other patients reporting a positive answer to one of the 6 questions at the phone interview had been adequately assessed with Doppler ultrasound and/or chest CT angiography. (Table 3) Assuming all three unassessed swellings were due to undiagnosed VTE, the figures would change to 1.52% and 1.98% for the ASA and LMWH groups, respectively, which was still a nonsignificant difference (*p* = 0.78).

**Table 3:** Questions asked via telephone and proportion of positive responses

Question	Answer	ASA group 458 (60.2%)	LMWH group 303 (39.8%)
Did you experience any calf or thigh pain after surgery?	Yes	67 (14.6%)	30 (9.9%)
Did you experience any calf or thigh swelling after surgery?	Yes	41 (9.0%)	25 (8.3%)
Did you experience any chest pain episodes after surgery?	Yes	2 (0.4%)	1 (0.3%)
Did you experience any shortness of breath episodes after surgery?	Yes	2 (0.4%)	1 (0.3%)
Were you diagnosed with clot formation in either of your legs after surgery?	Yes	4 (0.9%)	5 (1.6%)
Were you diagnosed with lung embolism after surgery?	Yes	2 (0.4%)	1 (0.3%)
Did you have any bleeding from/around the wound requiring medical attention after surgery?	Yes	19 (4.1%)	11 (3.6%)
Did you have any abnormal bleeding in other areas of the body requiring medical attention after surgery?	Yes	0 (0%)	0 (0%)

Table 4 contains the findings of univariable (unadjusted) logistic regression models evaluating the impact of anticoagulant regimen, age, BMI, and gender on the likelihood of VTE, none being found to be a significant factor. More detailed information of the patients and their complication profile has been provided in the supplementary Tables 1 and 2.

**Table 4:** Univariate Logistic Regression Modeling for risk factors of VTE Development

		OR (CI %)	*P*-value
Age		0.99 (0.9–1.1)	0.85
BMI		1.0 (0.9–1.2)	0.80
Sex	Male	Ref.	0.90
	Female	0.9 (0.1–7.0)	
Anticoagulant	ASA	Ref.	0.51
	LMWHs	1.5 (0.4–5.3)	

n: number, %: Percentage, OR: odds ratio, CI: confidence interval, Ref: reference

## Discussion

In this retrospective cohort study, our investigation into the efficacy and safety of ASA versus LMWH for VTE prophylaxis following ACLR revealed no statistically significant difference. The study did not reject the null hypothesis, demonstrating no variance in the incidence of symptomatic VTE between the two groups. This lack of divergence in VTE rates was observed in 1.09% of patients who received ASA and 1.65% of those who received LMWH (*p* = 0.53), with an overall incidence of 1.31% within three months post-ACLR. Furthermore, our study did not identify any specific risk factors associated with the occurrence of VTE following ACLR in this group. Alongside this, we also observed no significant differences between the two groups concerning various complications, including wound discharge, wound infection, the need for I&D, deep knee infection, swelling, and hemarthrosis. The absence of a significant difference suggests that both ASA and LMWH may be equally viable options for VTE prophylaxis in the post-operative management of ACLR patients, especially in those with a low-risk profile. This equivalence offers clinicians the flexibility to choose between these anticoagulants based on patient-specific factors, such as tolerance and potential interactions with other medications, rather than efficacy in VTE prevention alone. For patients, this result implies that they might have more personalized prophylaxis options, potentially enhancing adherence to post-operative care plans and satisfaction with their treatment. However, our inability to detect significant differences between ASA and LMWH for VTE prophylaxis after ACLR may partially stem from the retrospective design’s limitations. The low incidence of VTE in our cohort, in addition to the initial exclusion of patients with known risk factors for VTE, constrained our ability to detect pinpoint further risk factors. This situation raises the risk of type II errors, suggesting that our study might not have been sufficiently powered to capture existing efficacy differences between the prophylaxis strategies. These insights underscore the need for future, well-designed prospective research to accurately evaluate these treatments.

### Effectiveness, safety

In two articles with the highest level of evidence, chemoprophylaxis demonstrated its superiority in VTE reduction. A meta-analysis of eight randomized studies, providing Level 1 evidence and involving 4,113 knees, revealed that LMWH significantly decreased the risk of VTE after ACLR compared to no prophylaxis (RR = 0.22, *p* = 0.01) without increasing the risk of major bleeding (RR = 1.80, *p* = 0.61) or hematoma formation [16]. Furthermore, a randomized controlled trial demonstrated that extended-duration post-discharge thromboprophylaxis with enoxaparin, administered in an outpatient setting, significantly reduced the incidence of DVT in ACL surgery patients, compared with enoxaparin limited to in-hospital thromboprophylaxis, without increasing major or minor bleeding [17]. While there is substantial evidence supporting the safety of a no-prophylaxis policy after simple knee arthroscopy procedures in low-risk patients, the role of thromboprophylaxis after ACLR is more strongly emphasized than in simple knee arthroscopy [10, 16, 17, 28, 29]. However, it is essential to acknowledge that significant debate and controversy persist in this area, and there is limited evidence regarding the relative effectiveness of various anticoagulants in this specific clinical scenario [30].

Our analysis demonstrates that various other complications, including wound discharge, wound infection, the need for I&D, deep knee infection, as well as swelling and hemarthrosis, do not exhibit significant differences between the two groups. These findings align with prior research [16, 17, 31, 32]. However, owing to the absence of a control group, our study refrains from offering specific recommendations concerning the safety or effectiveness of routine thromboprophylaxis usage.

Furthermore, inflammatory biomarkers like neutrophil-lymphocyte ratio, CRP, IL-6, and D-dimer, associated with elevated cardiovascular risk and mortality in various conditions, may influence antithrombotic therapy outcomes by affecting platelet function and the balance between bleeding and thrombosis [33–39]. Despite their potential implications, the clinical application and significance of these biomarkers in the context of ACLR and chemoprophylaxis remain underexplored, necessitating further research to elucidate their role and impact.

### Previous studies, what are their findings?

A comparison of our results with prior studies provides valuable context. A study by Schmitz et al., which encompassed 26,014 ACLR cases with various thromboprophylaxis regimens, reported a symptomatic VTE incidence of 0.4%, but it did not analyze the correlation between thromboprophylaxis type and VTE incidence [6]. Similarly, McIntire et al. investigated 1,233 patients post-ACLR, with 821 receiving no chemoprophylaxis and 412 receiving aspirin (325 mg/day). In their study, only 0.8% of patients developed symptomatic DVT, with no significant difference observed between the group receiving chemoprophylaxis and the one without (*p* = 0.91) [40]. However, this study did not exclude individuals undergoing concurrent surgical procedures or revision surgeries, factors known to prolong surgery duration and elevate VTE risk, potentially influencing outcomes. Our study, in contrast, specifically excluded such cases to maintain a truly low-risk cohort. In a separate study, a 0% incidence of VTE was reported in low-risk patients following knee arthroscopy, which included ACLR cases, with some individuals receiving aspirin prophylaxis. However, the absence of observed VTE events in this study might be attributed to its small sample size of 170. Furthermore, the inclusion of elderly participants, such as those aged 75 years, challenges the characterization of the study cohort as uniformly low risk [19]. Consequently, our documented VTE incidence of 1.31% provides valuable insights into the VTE risk among a specific low-risk ACLR population, thereby filling an important gap in the existing literature.

However, several studies emphasize the importance of chemoprophylaxis in high-risk groups. Gaskill et al., after examining 16,558 ACLR cases, reported an overall VTE risk of 0.52%, with an elevated VTE risk identified in patients who smoked, were of advanced age, used anticoagulants, or underwent concurrent procedures such as high tibial osteotomy or posterior collateral ligament reconstruction [3]. Bokshan et al., who analyzed 9,146 individuals following ACLR, found a DVT incidence of 0.5% and identified age over 30 years, concurrent high tibial osteotomy or microfracture, and wound infection as risk factors for VTE, advocating for thromboprophylaxis in these subgroups [5]. Additionally, a systematic review by Janssen et al. in 2016 reported a DVT incidence of 9.7% after ACLR without thromboprophylaxis, with 2.1% being symptomatic. Their recommendation was to provide thromboprophylaxis treatment for ACLR patients with a moderate to high risk of VTE [4]. While the rate of VTE was relatively low in our study and in other similar studies, its significant clinical importance should not be underestimated.

### Various recommendations

In the most recent ICM, while the consensus discouraged the use of chemical prophylaxis after ACLR, it is essential to note that the level of recommendation for this practice remains at a low to moderate level. The ICM also highlighted the uncertainty surrounding the comparative safety and effectiveness of different pharmacologic agents, thus indicating the need for individualized risk stratification based on factors such as medical comorbidities, weight-bearing status, and immobilization when considering pharmacologic thromboprophylaxis for patients undergoing ACLR [18]. 

Conversely, as noted in the recent ICM, there exist varied recommendations for thromboprophylaxis following arthroscopic knee surgery across different countries [18]. For instance, the French Society of Anesthesiology and Intensive Care advocates for pharmacologic prophylaxis post-arthroscopic knee surgery [20]. Routine thromboprophylaxis involving anticoagulants is commonly practiced by most surgeons in Germany for outpatient arthroscopic knee procedures [21]. Furthermore, insights from a recent expert panel, comprising representatives from diverse countries, indicated that approximately one-third of the panel supported prophylaxis in single ligament reconstruction procedures [10]. In studies with the highest level of evidence, chemoprophylaxis has exhibited superiority in reducing VTE. To our knowledge, not many high-level evidence studies have discouraged routine thromboprophylaxis after ACLR. Therefore, establishing definitive recommendations, whether in favor of or against its routine use, presents a challenge. The divergent opinions could explain the differing global recommendations for thromboprophylaxis across various medical centers and countries.

Our study cannot make a recommendation for or against the use of chemoprophylaxis in this specific patient group, as we did not have a control group of no-thromboprophylaxis. However, it offers valuable insights for surgeons who use chemoprophylaxis in this specific patient population. It serves as evidence that, within this population, complications and the effectiveness in preventing VTE may be comparable between these two drugs.

### Need for further studies

It is important to acknowledge that retrospective studies on thromboprophylaxis after arthroscopic ACLR may exhibit a high selection bias, as patients at a higher risk of VTE were more likely to receive anticoagulant agents. Consequently, this potential type II error may lead to an underestimation of the effect of thromboprophylaxis. Therefore, future prospective and randomized studies are essential to provide more reliable evidence about effectiveness, safety and necessity.

### Strengths of the study

Our study adds new information on the optimal prophylactic regimen in ACLR patients, as there are no comparative studies on LMWH with aspirin and the available RCTs support routine chemical thromboprophylaxis [10, 19–21]. However, it has a small sample size and only 10 events, which limits our ability to compare effectiveness or identify risk factors with confidence. We acknowledge this as a major limitation and suggest further studies with larger samples. Our study also has minimal selection bias in terms of baseline risk for VTE development, as the baseline demographic parameters were similar between the groups, except for a small difference in mean age (29.27 ± 8.40 vs. 30.49 ± 7.12, for ASA vs. LMWH groups, respectively).

While the difference in mean age between groups was statistically significant, a difference of 1.2 years is unlikely to have a clinically significant effect on the rate of VTE [9].

### Limitations of the study

The study’s retrospective design introduces potential data inaccuracies and lacks a sample size calculation or systematic follow-up for all patients. Specifically, the patients’ comorbidities at the time of surgery were not always fully documented in the charts and were therefore reassessed through phone call interviews. This might have caused some inaccuracies due to recall bias. However, the findings regarding the main outcome of the study, VTE events, were more reliable, since the recorded data closely matched the phone interview findings. The study’s focus on leg swelling and pain as indicators of symptomatic DVT might not have encompassed all DVT symptoms, limiting a comprehensive understanding. Relying on yes/no responses for these symptoms lacks specificity, making it challenging to distinguish postoperative manifestations from potential signs of thrombosis. However, leg pain and swelling are the most sensitive symptoms of DVT [41–43]. Likewise, the inquiry into the presence of noticeable leg swelling was one of the patient-reported questions. Given the subjective nature of this sensation, its accuracy might have been limited.

Also, the lack of access to some data such as the length of surgery might have hidden some potential sources of bias. Due to the specificity of our research question and population of interest, our findings are not generalizable to all patients undergoing ACLR. This includes other graft types, ethnicities, and high-risk patients for VTE. Previous studies have not shown significant differences in VTE tendency between Middle Eastern and white ethnicities, but further research is needed to confirm this [44]. The fact that each group of patients was operated by a different surgeon could have introduced some selection bias. However, both surgeons used a similar surgical technique and postoperative protocol. Also, the lack of knee function assessments in our study represents a limitation.

## Conclusion

This study on isolated anterior cruciate ligament reconstruction in patients with a low baseline risk for venous thromboembolism showed that aspirin is as effective as low molecular weight heparins for VTE prophylaxis after this surgery. Thus, ASA, an affordable, accessible, and simpler-to-use agent compared to LMWH, could be safely used for low-risk ACLR patients. A prospective study to compare ASA, LMWH and no-prophylaxis is warranted to confirm the findings and also the necessity of VTE prophylaxis after ACLR in low-risk patients.

### Electronic supplementary material

Below is the link to the electronic supplementary material.

Supplementary Material 1

## Data Availability

The datasets generated during the current study are available from the corresponding author upon reasonable request.

## References

[1] Mall NA, Chalmers PN, Moric M, Tanaka MJ, Cole BJ, Bach BR, Paletta GA (2014). Incidence and trends of anterior cruciate ligament reconstruction in the United States. Am J Sports Med.

[2] Maletis GB, Inacio MC, Funahashi TT (2013). Analysis of 16,192 anterior cruciate ligament reconstructions from a community-based registry. Am J Sports Med.

[3] Gaskill T, Pullen M, Bryant B, Sicignano N, Evans AM, DeMaio M (2015). The prevalence of symptomatic deep venous thrombosis and pulmonary Embolism after Anterior Cruciate Ligament Reconstruction. Am J Sports Med.

[4] Janssen RP, Reijman M, Janssen DM, van Mourik JB (2016). Arterial complications, venous thromboembolism and deep venous thrombosis prophylaxis after anterior cruciate ligament reconstruction: a systematic review. World J Orthop.

[5] Bokshan SL, DeFroda SF, Panarello NM, Owens BD (2018). Risk factors for deep vein thrombosis or pulmonary Embolus following anterior Cruciate Ligament Reconstruction. Orthop J Sports Med.

[6] Kraus Schmitz J, Lindgren V, Janarv P, Forssblad M, Stålman A (2019). Deep venous thrombosis and pulmonary embolism after anterior cruciate ligament reconstruction: incidence, outcome, and risk factors. Bone Joint J.

[7] Traven SA, Farley KX, Gottschalk MB, Goodloe JB, Woolf SK, Xerogeanes JW, Slone HS (2021). Combined oral contraceptive use increases the risk of venous thromboembolism after knee arthroscopy and Anterior Cruciate Ligament Reconstruction: an analysis of 64,165 patients in the Truven database. Arthroscopy.

[8] Reynolds AW, Garay M, Lynch S, Black KP, Gallo RA (2022). Incidence of venous thromboembolism following knee arthroscopy: effectiveness of a risk-based stratified Chemoprophylaxis Protocol. J Knee Surg.

[9] Nagashima MOT, Takeshima K, Seki H, Nakayama M, Origuchi N, Ishii K (2020). Unexpectedly high incidence of venous thromboembolism after arthroscopic anterior cruciate ligament reconstruction: prospective, observational study. J ISAKOS.

[10] Easwaran R, Khan M, Sancheti P, Shyam A, Bhandari M, Ranawat AS, Thakkar S, Parikh S, Musahl V, Joglekar S (2022). Prophylaxis for preventing venous thromboembolism in knee arthroscopy and soft tissue reconstruction: consensus statements from an international panel of experts. Knee Surg Sports Traumatol Arthrosc.

[11] Cancienne JM, Diduch DR, Werner BC (2017). High Altitude is an independent risk factor for postoperative symptomatic venous thromboembolism after knee arthroscopy: a matched case-control study of Medicare patients. Arthroscopy.

[12] Holler JT, Salesky M, Halvorson RT, Zhang AL, Ma CB, Feeley BT, Leavitt AD, Colyvas N, Lansdown DA. Perioperative Thromboprophylaxis Is Associated With Lower Risk of Venous Thromboembolism After Knee Arthroscopy. *Arthroscopy* 2022. 10.1016/j.arthro.2022.06.034 35840070

[13] Sun Y, Chen D, Xu Z, Shi D, Dai J, Qin J, Jiang Q (2014). Incidence of symptomatic and asymptomatic venous thromboembolism after elective knee arthroscopic surgery: a retrospective study with routinely applied venography. Arthroscopy.

[14] Tyson JJ, Bjerke BP, Genuario JW, Noonan TJ (2016). Thromboembolic events after arthroscopic knee surgery: increased risk at high elevation. Arthroscopy.

[15] Keller RA, Moutzouros V, Dines JS, Bush-Joseph CA, Limpisvasti O (2018). Deep venous thrombosis Prophylaxis in Anterior Cruciate Ligament reconstructive surgery: what is the current state of practice?. Sports Health.

[16] Zhu J, Jiang H, Marshall B, Li J, Tang X (2019). Low-molecular-weight heparin for the Prevention of venous thromboembolism in patients undergoing knee arthroscopic surgery and Anterior Cruciate Ligament Reconstruction: a Meta-analysis of Randomized controlled trials. Am J Sports Med.

[17] Marlovits S, Striessnig G, Schuster R, Stocker R, Luxl M, Trattnig S, Vécsei V (2007). Extended-duration Thromboprophylaxis with Enoxaparin after arthroscopic surgery of the anterior cruciate ligament: a prospective, randomized, placebo-controlled study. Arthroscopy: J Arthroscopic Relat Surg.

[18] Recommendations from the ICM-VTE (2022). Sports. J Bone Joint Surg Am.

[19] Kaye ID, Patel DN, Strauss EJ, Alaia MJ, Garofolo G, Martinez A, Jazrawi LM. Prevention of Venous Thromboembolism after Arthroscopic Knee Surgery in a Low-Risk Population with the Use of Aspirin. A Randomized Trial. *Bull Hosp Jt Dis (*2013*)* 2015, 73(4):243–248. 26630467

[20] Samama CM, Gafsou B, Jeandel T, Laporte S, Steib A, Marret E, Albaladejo P, Mismetti P, Rosencher N (2011). [French Society of Anaesthesia and Intensive Care. Guidelines on perioperative venous thromboembolism prophylaxis. Update 2011. Short text]. Ann Fr Anesth Reanim.

[21] Müller-Rath R, Ingenhoven E, Mumme T, Schumacher M, Miltner O (2010). Perioperatives Management in Der Ambulanten Arthroskopischen Chirurgie Des Kniegelenks. Z Orthop Unfall.

[22] Grant PJ, Greene MT, Chopra V, Bernstein SJ, Hofer TP, Flanders SA (2016). Assessing the Caprini score for Risk Assessment of venous thromboembolism in Hospitalized Medical patients. Am J Med.

[23] Diagnosis. and Treatment of Venous Thromboembolism [https://www.cdc.gov/ncbddd/dvt/diagnosis-treatment.html].

[24] 2019 ESC Guidelines for the Diagnosis and Management of Acute PE. [https://www.acc.org/latest-in-cardiology/articles/2020/07/10/08/44/2019-esc-guidelines-for-the-diagnosis-and-management-of-acute-pe].

[25] Stone J, Hangge P, Albadawi H, Wallace A, Shamoun F, Knuttien MG, Naidu S, Oklu R (2017). Deep vein thrombosis: pathogenesis, diagnosis, and medical management. Cardiovasc Diagn Ther.

[26] Groenwold RHH, Dekkers OM. Is it a risk factor, a predictor, or even both? The multiple faces of multivariable regression analysis. Eur J Endocrinol 2023, 188(1). 10.1093/ejendo/lvac012 36651166

[27] Heinze G, Wallisch C, Dunkler D (2018). Variable selection - A review and recommendations for the practicing statistician. Biom J.

[28] van Adrichem RA, Nemeth B, Algra A, le Cessie S, Rosendaal FR, Schipper IB, Nelissen R, Cannegieter SC (2017). Thromboprophylaxis after knee arthroscopy and Lower-Leg casting. N Engl J Med.

[29] Perrotta C, Chahla J, Badariotti G, Ramos J (2020). Interventions for preventing venous thromboembolism in adults undergoing knee arthroscopy. Cochrane Database Syst Rev.

[30] Forlenza EM, Parvaresh KC, Cohn MR, Lavoie-Gagne O, Khazi ZM, Lu Y, Cregar W, Forsythe B (2022). Incidence and risk factors for symptomatic venous thromboembolism following anterior cruciate ligament reconstruction. Knee Surg Sports Traumatol Arthrosc.

[31] Sun Y, Chen D, Xu Z, Shi D, Dai J, Qin J, Qin J, Jiang Q (2014). Deep venous thrombosis after knee arthroscopy: a systematic review and meta-analysis. Arthroscopy.

[32] Chapelle C, Rosencher N, Jacques Zufferey P, Mismetti P, Cucherat M, Laporte S (2014). Prevention of venous thromboembolic events with low-molecular-weight heparin in the non-major orthopaedic setting: meta-analysis of randomized controlled trials. Arthroscopy.

[33] Ding J, Yue X, Tian X, Liao Z, Meng R, Zou M (2023). Association between inflammatory biomarkers and venous thromboembolism: a systematic review and meta-analysis. Thromb J.

[34] Hu J, Cai Z, Zhou Y (2022). The Association of Neutrophil–lymphocyte ratio with venous thromboembolism: a systematic review and Meta-analysis. Clin Appl Thromb Hemost.

[35] Seo WW, Park MS, Kim SE, Lee JH, Park DG, Han KR, Oh DJ, Hyon MS (2021). Neutrophil-lymphocyte ratio as a predictor of venous thromboembolism after total knee replacement. J Knee Surg.

[36] Phan T, Brailovsky Y, Fareed J, Hoppensteadt D, Iqbal O, Darki A (2020). Neutrophil-to-lymphocyte and platelet-to-lymphocyte ratios Predict all-cause mortality in Acute Pulmonary Embolism. Clin Appl Thromb Hemost.

[37] Gong P, Liu Y, Gong Y, Chen G, Zhang X, Wang S, Zhou F, Duan R, Chen W, Huang T (2021). The association of neutrophil to lymphocyte ratio, platelet to lymphocyte ratio, and lymphocyte to monocyte ratio with post-thrombolysis early neurological outcomes in patients with acute ischemic stroke. J Neuroinflamm.

[38] Efe E, Kocayiğit I, Türker PM, Murat K, Erkan A, Sedat T, Alper Ç, Necati AM, Gökhan VM, Bahri A (2016). Platelet-to-lymphocyte ratio but not neutrophil-to-lymphocyte ratio predicts high on-treatment platelet reactivity in clopidogrel-treated patients with acute coronary syndrome. Indian J Pharmacol.

[39] Santoro L, Ferraro PM, Nesci A, D’Alessandro A, Macerola N, Forni F, Tartaglione R, De Vitis R, Gasbarrini A, Santoliquido A (2021). Neutrophil-to-lymphocyte ratio but not monocyte-to-HDL cholesterol ratio nor platelet-to-lymphocyte ratio correlates with early stages of lower extremity arterial disease: an ultrasonographic study. Eur Rev Med Pharmacol Sci.

[40] McIntire SC, Bernstein EM, Tompane TM, Briggs AM, Ferris WJ, Renninger CH, McDonald LS, Hurvitz AP (2021). Aspirin for deep-venous thrombosis Prophylaxis after Anterior Cruciate Ligament Reconstruction. Mil Med.

[41] Sandler DA, Martin JF, Duncan JS, Blake GM, Ward P, Ramsay LE, Lamont AC, Ross B, Sherriff S, Walton L (1984). Diagnosis of deep-vein thrombosis: comparison of clinical evaluation, ultrasound, plethysmography, and venoscan with X-ray venogram. Lancet.

[42] Chopard R, Albertsen IE, Piazza G (2020). Diagnosis and treatment of lower extremity venous thromboembolism: a review. JAMA.

[43] Bikdeli B, Caraballo C, Trujillo-Santos J, Galanaud JP, di Micco P, Rosa V, Cusidó GV, Schellong S, Mellado M, Del Valle Morales M (2022). Clinical presentation and short- and long-term outcomes in patients with isolated distal deep vein thrombosis vs proximal deep vein thrombosis in the RIETE Registry. JAMA Cardiol.

[44] Lazo-Langner A, Liu K, Shariff S, Garg AX, Ray JG (2018). Immigration, region of origin, and the epidemiology of venous thromboembolism: a population-based study. Res Pract Thromb Haemost.

