# Supplementary material for: Aspirin is as effective as low molecular weight heparins in preventing symptomatic venous thromboembolism following arthroscopic anterior cruciate ligament reconstruction
Source: BMC Musculoskelet Disord. 2024 Feb 19;25:154. doi: 10.1186/s12891-024-07282-8 (PMC10875785; doi:10.1186/s12891-024-07282-8)
Supplement: Supplementary file 1 — Supplementary Material 1 [file 12891_2024_7282_MOESM1_ESM.docx]

Supplementary Table 1s. Baseline characteristics of the patients according to the groups.

|  |  | Aspirin | LMWH | P-value |
| --- | --- | --- | --- | --- |
|  | | 474 (60%) | 316 (40%) |  |
| Sex | **Female** | 58 (12.2%) | 30 (9.5%) | 0.25 |
|  | **Male** | 416 (87.8%) | 286 (90.5%) |  |
| Age | | 29.60 (8.8) | 30.56 (7.2) | 0.10 |
| BMI | | 25.10 (3.6) | 25.45 (3.7) | 0.20 |
| Smoke | **Yes** | 52 (11%) | 33 (10.4%) | 0.90 |
| DM | **Yes** | 4 (0.8%) | 2 (0.6%) | 0.74 |
| IBD | **Yes** | 1 (0.2%) | 3 (1%) | 0.31 |
| Active cancer | **Yes** | 1 (0.2%) | 0 | 0.41 |
| DVT History | **Yes** | 0 | 1 (0.3%) | 0.40 |
| Pulmonary disease | **Yes** | 5 (1.0%) | 6 (1.9%) | 0.36 |
| Cardiac disease | **Yes** | 6 (1.3%) | 3 (1%) | 0.75 |
| Injury Cause | **Sport** | 428 (90.3%) | 274 (86.7%) | 0.40 |
|  | **Falling** | 29 (6.1%) | 24 (7.6%) |  |
|  | **Accident** | 13 (2.8%) | 13 (4.1%) |  |
|  | **Direct Trauma** | 4 (0.8%) | 5 (1.6%) |  |

Categorical- and Continuous variables are presented in *number (percent)* and *mean (standard deviation)* formats, respectively.

Supplementary Table 2s. Complication profile.

|  | |  | Aspirin | LMWH | P-value |
| --- | --- | --- | --- | --- | --- |
| Treatment | | | 474 (60%) | 316 (40%) |  |
| VTE | **DVT** | | 3 (0.6%) | 4 (1.3%) | 0.72 |
|  | **PTE** | | 1 (0.2%) | 0 |  |
|  | **Both** | | 1 (0.2%) | 1 (0.3%) |  |
|  | **Total VTE events** | | 5 (1.0%) | 5 (1.6%) | 0.53 |
| Other Complications | **Wound Discharge** | | 23 (4.8%) | 15 (4.7%) | 0.95 |
|  | **Wound Infection** | | 15 (3.2%) | 9 (2.8%) | 0.84 |
|  | **Need for I&D** | | 5 (1.0%) | 8 (2.5%) | 0.15 |
|  | **Deep Knee Infection** | | 4 (0.8%) | 7 (2.2%) | 0.13 |
|  | **Swelling & Hemarthrosis** | | 20 (4.2%) | 13 (4.1%) | 0.94 |

VTE: venous thromboembolism, PTE: pulmonary thromboembolism, I&D: irrigation & debridement.

Events are presented as *numbers (percentages).*

Supplementary Table 3s. Baseline characteristics of the patients according to VTE event.

|  |  | VTE | No VTE | P-value |
| --- | --- | --- | --- | --- |
| Anticoagulant | ASA | 5 (50%) | 469 (60.1%) | 0.53 |
|  | Enoxaparin | 5 (50%) | 311 (39.9%) |  |
| Sex | Female | 1 (10.0%) | 87 (11.1%) | 0.91 |
|  | Male | 9 (90.0%) | 693 (88.8%) |  |
| Age(y) | | 29.3**±** 6.3 | 29.99**±** 8.2 | 0.79 |
| BMI(Kg/m2) | | 25.5**±** 3.0 | 25.45**±** 6.9 | 0.98 |
| smoke | yes | 0(00.0%) | 85 (10.9%) | 0.61 |
|  | no | 10 (100.0%) | 695 (89.1%) |  |
| DM | yes | 0 (00.0%) | 6 (0.8%) | 0.78 |
|  | no | 10 (100.0%) | 774 (99.2%) |  |
| IBD | yes | 0 (00.0%) | 4 (0.5%) | 0.82 |
|  | no | 10 (100.0%) | 776 (99.5%) |  |
| Active cancer | yes | 0 (00.0%) | 1 (0.1%) | 0.91 |
|  | no | 10 (100.0%) | 779 (99.87%) |  |
| DVT History | yes | 0 (00.0%) | 1 (0.1%) | 0.91 |
|  | no | 10 (100.0%) | 779 (99.9%) |  |
| OCP | yes | 0 (00.0%) | 0 (00.0%) | - |
|  | no | 10 (100.0%) | 780 (100.0%) |  |
| Pulmonary HTN | yes | 0 (00.0%) | 3 (0.4%) | 0.84 |
|  | no | 10 (100.0%) | 777 (99.6%) |  |
| Pulmonary disease | yes | 0 (00.0%) | 8 (1.0%) | 0.75 |
|  | no | 10 (100.0%) | 772 (99.0%) |  |
| Cardiac disease | yes | 0 (00.0%) | 9 (1.1%) | 0.73 |
|  | no | 10 (100.0%) | 771 (98.9%) |  |

Categorical- and Continuous variables are presented in *number (percent)* and *mean (standard deviation)* formats, respectively. y: years, Kg: weight in kilograms, m: height in meters

Supplementary Table 4s. Complication profile according to VTE event.

|  | |  | VTE | No VTE | P-value |
| --- | --- | --- | --- | --- | --- |
| Complications | **Wound Discharge** | | 2 (20.0%) | 36 (4.6%) | 0.08 |
|  | **Wound Infection** | | 0 (0.0%) | 24 (3.1%) | 0.57 |
|  | **Need for I&D** | | 0 (0.0%) | 13 (1.7%) | 0.68 |
|  | **Deep Knee Infection** | | 0 (0.0%) | 11 (1.4%) | 0.70 |
|  | **Swelling & Hemarthrosis** | | 2 (4.0%) | 31 (20.0%) | 0.06 |

Abbreviations are as the following: n number, and % Percentage
